# Air–Bone Gap in Meniere’s Disease: A Case Series and Literature Review

**DOI:** 10.3390/audiolres14060081

**Published:** 2024-11-08

**Authors:** Arun Pajaniappane, Nilesh Parekh, Anita Wong

**Affiliations:** 1 Harley Street Audiovestibular Clinic, London W1G 7HP, UK; nilesh.parekh@harleyavm.com (N.P.); a.wong@harleyavm.com (A.W.); 2 St George’s University Hospitals NHS Foundation Trust, London SW17 0QT, UK; 3 Great Ormond Street Hospital for Children NHS Foundation Trust, London WC1N 3BH, UK

**Keywords:** conductive hearing loss, Meniere’s disease, third window syndromes

## Abstract

Background/Objectives: An air–bone gap (ABG) on audiometry is usually secondary to a conductive hearing loss. However, persistent and repeatable ABGs on audiometry in the absence of external or middle ear pathology is thought to arise from inner ear disorders including Meniere’s Disease (MD). In this paper, we aim to showcase this interesting finding occurring in MD with an associated literature review. Methods: Using retrospective review and analysis of case notes, we describe eight cases of persistent ABG on audiometry in MD. All other causes for the ABG were explored and excluded with the aid of objective audiological testing. Results: ABG can occur in a small sub-set of the MD population. In our case series, the ABG was typically found to affect the low frequencies. Higher frequencies appear to be spared. However, a detailed history, examination and battery of objective tests are required to ensure that all other causes of the ABG are reliably considered and excluded prior to attributing it to the inner ear. The finding of an ABG in MD may be dependent on the stage of the disease. Further research is required to determine the underlying cause of the ABG and its potential applications to help guide treatment.

## 1. Introduction

Greater air conduction thresholds compared to bone conduction thresholds on pure-tone audiometry (PTA) is known as an ABG. When the difference between air and bone conduction thresholds at a frequency is greater than 15 dBHL, it is considered to be significant [1].

The identification of a consistent and persistent ABG on audiometry can usually be ascribed to a conductive or mixed hearing loss. Usually, the source of the conductive component can be localised to an external or middle ear lesion such as cerumen, middle ear effusion, tympanic membrane perforation or otosclerosis, amongst others. However, unexplained conductive hearing loss with no obvious middle ear pathology was recognised and first described as early as 1980 [2]. The ABG was typically noted to affect the low frequencies.

This phenomenon was also identified in other studies of middle ear conditions where no obvious pathology was identified, and the term “inner ear conductive hearing loss” came into usage [3]. Other terms used include pseudo-conductive hearing loss [4] or false conductive loss.

Improved recognition and further studies have provided greater insight into the inner ear mechanisms resulting in an ABG on audiometry [5]. This can be due to a combination of improved bone conduction thresholds [4] alongside a reduction in air conduction thresholds [6]. Conditions typically thought to present with an inner ear ABG include enlarged vestibular aqueducts [7,8], Superior Semi-circular Canal Dehiscence (SSCD) [6] and perilymph fistula [4]. Other conditions linked to an ABG are x-linked stapes gusher syndrome [9] and dural arterio-venous fistula [10]. The proposed mechanism of the ABG in these cases has been hypothesised to be secondary to the presence of a pathologic third window, which results in the dissipation of acoustic energy [6].

An inner ear ABG has also been described in MD in the absence of any obvious external or middle ear pathology. MD is a chronic condition presenting with episodic vertigo, fluctuating hearing loss and tinnitus. It has been shown to affect about 190 in 100,000, but some studies have indicated a prevalence as high as 513 per 100,000 [11]. Prevalence increases with age [12].

The cadaveric study of human temporal bones [13] first demonstrated endolymphatic hydrops as the likely underlying pathology resulting in the clinical syndrome of MD. This was eventually confirmed in a comprehensive review [14]. More recent imaging methods have demonstrated hydrops in vivo, leading to greater diagnostic accuracy [15].

Endolymphatic hydrops is the distention of endolymphatic spaces secondary to a pathological increase in endolymph fluid [16]. It results in the clinical syndrome of MD, which presents with episodic audiovestibular symptoms, leading to progressive vestibulo-cochlear damage. Typically, this results in a progressive sensorineural hearing loss, which is more pronounced in the lower and mid-frequencies [17].

In a study of 40 MD patients published in 1989, 32.5% (*n* = 13) of patients were identified to have an ABG predominantly affecting the low frequencies [18]. It is worth noting that this study was limited by being conducted at a time when knowledge of Third Mobile Window Disorders (TMWDs) and their association with ABG on audiometry was still emerging. As a result, TMWDs may not have been consistently or reliably ruled out.. Few subsequent studies have explored this phenomenon in further detail. A Turkish study in 2005 of 84 MD patients identified a similar pattern, with 28.4% (*n* = 24) of their cohort demonstrating an ABG on at least one frequency, with an average gap of 11.5 dB [19]. A retrospective analysis of 337 patients with definite MD showed the prevalence of low-frequency ABGs in 13.9% of their study population [20]. A study of 35 patients with unilateral definite MD showed ABG affecting low frequencies in 28.6% of their cohort [21]. Delayed post gadolinium MRI features in this sub-set were compared to patients without an ABG.

All studies suggest that the mechanism of the ABG in MD is different to the ABG arising secondary to the pathologic third window found in SSCD and similar conditions. It was postulated that endolymphatic hydrops exerts pressure onto the medial aspect of the stapes footplate, dampening footplate mobility and resulting in an ABG [18,19]. Evidence from the gadolinium enhanced study suggests that direct contact of the distended saccule with the stapes footplate is the source of the ABG [21].

## 2. Materials and Methods

In this paper, we aim to present examples of low-frequency ABGs from our specialist practice, with a focus on MD, as this showcases a unique phenomenon which so far has only been partly described.

This study was conducted at a busy quaternary-level central London private clinic, dedicated to seeing patients with hearing and balance disorders. A retrospective case review of clinical notes, clinical correspondence, imaging, audiological and vestibular test results was performed over a period from 2023 to 2024. Cases notes were identified on local electronic patient records using the search terms “conductive”, “endolymphatic hydrops” and “Meniere’s disease”.

The cases identified from the initial search were reviewed by the lead author, an Audiovestibular Medicine Physician, to confirm that they met the Barany Society’s criteria for diagnosis of either definite or probable MD [17]. From this cohort, only cases demonstrating hearing loss with an ABG at one or more frequencies within the 0.5 to 4 kHz range were selected. 

Patients were only included if the lead author confirmed that other conditions that may cause an ABG, such as external or middle ear pathology and TMWDs, have been reliably excluded through appropriate clinical examination and a battery of objective testing.

The clinical examination must have documented a normal ear examination, specifically noting the absence of cerumen or perforation, for the case to be selected.

Diagnostic testing for the auditory system included pure-tone audiometry (PTA), tympanometry, stapedial acoustic reflex thresholds (ARTs) and otoacoustic emissions (OAEs) testing. Testing was performed using a calibrated Bio-logic^®^ AuDX Pro Flex^®^ with Sennheiser HDA 300 supra-aural headphones and a RadioEar B71 bone conductor, with EP-TY 9302851 and EP-DP 5300474 probes for ART and OAE testing, sourced in London, United Kingdom. Testing was performed in a controlled sound-proof booth in a quiet clinical environment.

PTA was performed as per British Society of Audiology (BSA) guidelines [22], at the frequency ranges from 0.25 kHz to 8 kHz for air conduction. Bone conduction thresholds were performed at 0.5 kHz to 2 kHz. The 4 kHz bone conduction was often not performed as per guidelines due to the possibility of false ABGs. The ABG was calculated and analysed at the frequencies 0.5, 1 and 2 kHz.

Tympanometry was performed using a 226 Hz stimulus, and ipsilateral ARTs were measured at 1 and 4 kHz. Both the distortion product and transient-evoked OAEs were performed to assess outer hair cell function.

The following possible sources of variability were assessed for and controlled: The audiometer was calibrated in line with BSA standards [22], with a full annual calibration in addition to stage A calibration performed daily. Individual test-to-test variation is minimised through training, auditing and having clear local protocols.

Tympanometry testing allowed for the exclusion of patients with tympanic membrane or middle ear pathology. ART and OAE testing were used to provide further diagnostic information to be used in conjunction with PTA and tympanometry results. For example, a present ART response with peaked tympanometry traces can help to determine the absence of ossicular chain pathology in hearing loss with an ABG.

If tympanometry or ART testing yielded abnormal results or were not performed, the patient was excluded from this study.

Follow-up hearing tests were also reviewed where available, to assess for any fluctuations over time. A simple descriptive analysis was performed to suitably describe the findings using Microsoft Excel.

For those patients who had specialist 3T MRI with delayed contrast, the test results were reviewed to confirm endolymphatic hydrops.

Diagnostic vestibular test battery performed to confirm vestibular dysfunction ipsilateral to the side of MD include the video head impulse test (vHIT), cervical vestibular-evoked myogenic potential (cVEMP), videonystagmography (VNG) and air caloric test. These were performed using Otometrics ICS Impulse for vHIT, Interacoustic Eclipse with insert headphones for cVEMP, Cyclops BalanceEye for VNG and Otometrics ICS AirCal for the caloric test.

cVEMP testing has been proposed to be a useful adjunct in the diagnosis of SSCD, with findings of high-amplitude responses and lower thresholds ipsilateral to the side of the lesion [23]. Any patients with these findings were hence also excluded from our case series. However, detailed analysis of vestibular testing was otherwise not considered as this is beyond the scope of this paper and was performed purely from the clinical diagnostic perspective.

## 3. Results

A total of 469 patient records were identified and reviewed from August 2023 to September 2024.

Eight patients were identified to meet the inclusion criteria within the study period. These patients satisfied the criteria of either definite or probable MD as per Barany Society’s criteria [17] and had active symptoms. They all had a significant ABG on PTA, with no obvious external or middle ear cause identified for the findings on clinical examination or a battery of objective tests. There were no symptoms of the Tullio phenomenon noted on the history.

There was an even distribution of male and female patients (four male, four female). The patients’ ages ranged from 29 to 66 years, with a mean age of 46 years, with a preponderance towards older age groups in keeping with previous studies [12]. Seven patients had unilateral MD, with four affecting the right ear, three patients with left MD and one patient with bilateral MD.

All patients (*n* = 8) had tympanometry and ART findings within normal limits. All patients underwent standard vestibular battery testing and demonstrated vestibular dysfunction on the same side as their MD. All underwent cVEMP testing with no indication of large-amplitude or low-threshold responses.

All eight patients underwent normal MRI imaging of the internal acoustic meatus (IAM). Four patients underwent 3T MRI imaging with delayed gadolinium contrast, which confirmed the clinical diagnosis of endolymphatic hydrops and correctly identified the affected side. [15].

**Figure 1:**
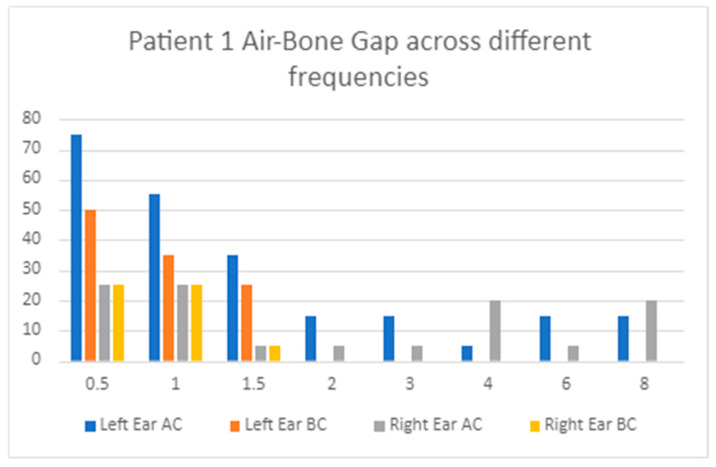
Visual representation of the ABG across all frequencies in one patient, demonstrating an ABG predominantly presenting unilaterally in the lower frequencies.

**Figure 2:**
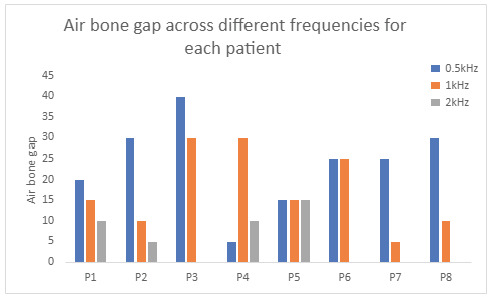
ABG is consistently present only at 0.5 kHz and 1 kHz across all eight patients.

Visual inspection and data analysis of audiometry results confirmed findings of the ABG predominantly affecting the low frequencies of the affected MD ear. Figure 1 provides a useful visual representation of the classical pattern of air and bone conduction thresholds across the range of frequencies in one patient. There is a low-frequency ABG from 0.5 kHz to 1.5 kHz. Higher frequencies appear to be spared or predominantly sensorineural. Figure 2 showcases this general pattern across all patients in our cohort, although ABG is only consistently present at 0.5 kHz and 1kHz.

**Figure 3:**
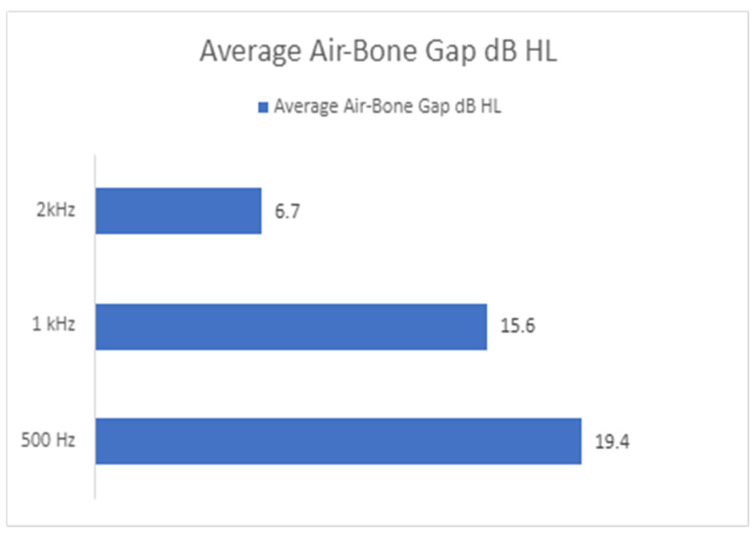
The average ABG gap across the low frequencies, demonstrating that 0.5 kHz and 1 kHz have the greatest difference.

Of the eight identified cases, the average ABG was greatest at 0.5 kHz, followed by 1 kHz. This was the case in six out of eight patients, with the other two patients having a larger ABG at 1 kHz. An ABG at 2 kHz was less common, occurring in only four of the cases and with a smaller difference, as demonstrated in Figure 3.

**Figure 4:**
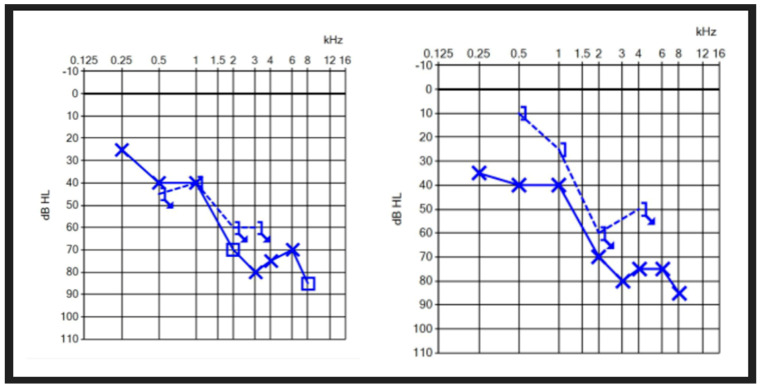
Audiometric changes over 1 month in a patient with active MD.

Of the eight patients, two underwent serial audiometry over the course of their active disease. Figure 4 showcases one patient where there was development of a new conductive component with a flare-up of MD, compared to the previously identified sensorineural hearing loss four weeks earlier. The testing conditions, equipment and tester were maintained, reducing the rate of inter-test and inter-tester variability.

## 4. Discussion

In this paper, we describe several cases of inner ear ABGs on audiometry in MD. Whilst fluctuating, low-frequency sensorineural hearing loss has been well described in the literature [17], only passing note is made of ABG, conductive or mixed hearing losses. Few studies have explored this phenomenon in detail despite the first description being as early as 1989 [18].

Our small cohort of MD patients were identified to have a significant low-frequency ABG on audiometry. The level of ABG on average was 19.4 dB at 0.5 kHz and 15.6 at 1 kHz. An ABG can also be present at 2 kHz, although inconsistently. High frequencies appear to be spared, although any ABG in this range should be viewed with caution due to the possibility of false conductive losses at 4 kHz. Clinical and objective assessment did not reveal any other obvious external or middle ear pathology to account for the identified ABG. In selected cases, the ABG was also seen to fluctuate and develop over time.

All patients in our cohort had a battery of audiovestibular testing to exclude other pathologies that may result in a conductive hearing loss, either true or pseudo-conductive.

Whilst outside the scope of this paper, it is important to note that the ABG is likely only present in a small sub-group of MD patients, as described in previous literature [18,19]. Whilst this phenomenon is unique and reproducible, it is worth exercising caution and ensuring that co-existent external and middle ear pathology or pathological third window has been reliably excluded. Hence, a battery of audiological and vestibular tests alongside relevant imaging is recommended to exclude other disorders.

The underlying pathophysiology of the ABG remains enigmatic but possibly related to pressure on the footplate secondary to hydrops, as suggested in previous papers [18,19]. The fluctuating nature of the conductive component seen in our cohort, as well as the increase in the ABG anecdotally noted during flare-ups of the disease, suggest that the increase in hydrostatic pressure [18] and secondary pressure on the footplate is the likely culprit.

Vestibular fibrosis has also previously been observed in temporal bone studies and the resulting connections to the footplate were thought to be a possible explanation for the Hennebert sign [24]. This may also result in an ABG. However, this explanation seems unlikely to be the cause due to the fluctuating element we noted in our limited case series.

As development of an ABG correlated with flare-ups of MD in some patients in our cohort, this can potentially be used as a marker to guide treatment. Previous studies have indeed identified a correlation between increased vertigo spells and development of an ABG [20]. However, greater patient numbers with serial audiometry and further larger scale studies are required to determine its usefulness as a clinical aid.

## 5. Conclusions

With this case series, we have been able to showcase the low-frequency ABG in MD in a limited population. The ABG in MD is an interesting yet under-researched phenomenon. As in previous studies and as confirmed by our series, it typically affects the low frequencies. The ABG was also noted to be fluctuant in nature, developing and settling over the course of the disease in some patients. It is worthwhile exploring this occurrence further, as it may provide clues to the mechanisms underlying endolymphatic hydrops and MD.

However, it is worth considering several limitations of this small pilot observational study, including low subject numbers. Whilst other conditions were diligently investigated, there remains the possibility that the ABG found in these patients can be due to an unidentified co-existing pathology. TMWDs can potentially have been overlooked and not picked up on cVEMP testing. Whilst all patients had an MRI IAM scan, a cone beam CT would be the imaging of choice to investigate for such conditions, but it is limited by unnecessary radiation exposure. Similarly, MRI IAM imaging may not pick up any subtle findings such as elevated intracranial pressure, which has been linked to the presence of an ABG.

Future studies should be considered with greater numbers to facilitate statistical analysis and assess the mathematical significance of the findings. The development of an ABG can also be analysed alongside symptom scores and serial audiometry, to better understand the correlation between the clinical picture and audiological findings. Clinical diagnosis of MD is based on history and audiological findings. However, it is worth ensuring that future studies are correlated with the findings of specialist 3T MRI imaging. Consideration of CT scans of the temporal bones may also be a useful adjunct when excluding other causes.

When considering an ABG in MD without any obvious external or middle ear pathology, other factors such as inter-test, inter-tester and environmental variability should also be considered first prior to attributing to an inner ear cause [25]. Subsequently, the cause of the inner ear ABG should be reliably investigated to attribute it to either a pathological third window effect or to MD. With a better understanding of an ABG in MD, it may serve as a useful adjunct to help guide treatment.

## Data Availability

The raw data supporting the conclusions of this article will be made available by the authors on request.
